# Inhibition of cancer cell growth by ruthenium complexes

**DOI:** 10.1186/s12967-016-0797-9

**Published:** 2016-02-12

**Authors:** Joji Iida, Elisabeth T. Bell-Loncella, Marc L. Purazo, Yifeng Lu, Jesse Dorchak, Rebecca Clancy, Julianna Slavik, Mary Lou Cutler, Craig D. Shriver

**Affiliations:** Department of Cell Biology, Windber Research Institute, 620 7th Street, Windber, PA 15963 USA; Department of Chemistry, University of Pittsburgh at Johnstown, 450 Schoolhouse Road, Johnstown, PA 15904 USA; Department of Pathology, Uniformed Services University for Health Sciences, 4301 Jones Bridge Road, Bethesda, MD 20184 USA; Department of Surgery, Walter-Reed National Military Medical Center, 8901 Rockville Pike, Bethesda, MD 20889 USA; Murtha Cancer Center, 8901 Rockville Pike, Bethesda, MD 20889 USA

**Keywords:** Ruthenium, Breast cancer, Growth, VFGF, GM-CSF, PDGF, Synergy, Anti-cancer reagents

## Abstract

**Background:**

Previous studies suggest that certain transition metal complexes, such as cisplatin, are efficacious for treating various cancer types, including ovarian, lung, and breast.

**Methods:**

In order to further evaluate ruthenium (Ru) complexes as potential anti-cancer agents, we synthesized and evaluated Ru-arene complexes. Two complexes with the general formula [Ru (*η*^6^-*p*-cym) (N–N) Cl]^+^ were tested for their abilities to inhibit cancer cells.

**Results:**

The complex with *o*-phenylenediamine as the N–N ligand (*o*-PDA) significantly inhibited growth of breast (MDA-MB-231, MCF-7, SKBR-3, and SUM149), lymphoma (Raji), melanoma (Bowes), and osteosarcoma (HT1080); however, the complex with *o*-benzoquinonediimine (*o*-BQDI) was ineffective except for SUM149. In contrast, *o*-PDA failed to inhibit growth of human breast epithelial cells, MCF-10A. Treatment of MDA-MBA-231 cells with *o*-PDA resulted in a significant reduction of productions of PDGF-AA, GM-CSF, and VEGF-A proteins at the transcriptional levels. Finally, we demonstrated that *o*-PDA synergistically inhibited MDA-MB-231 cell growth with cyclophosphamide but not doxorubicin or paclitaxel.

**Conclusion:**

These results suggest that Ru-arene complexes are promising anti-cancer drugs that inhibit progression and metastasis by blocking multiple processes for breast and other types of cancer.

**Electronic supplementary material:**

The online version of this article (doi:10.1186/s12967-016-0797-9) contains supplementary material, which is available to authorized users.

## Background

According to the latest issue of Cancer Facts and Figures (2015), more than 230,000 women will be diagnosed with invasive breast cancer in the USA and nearly 40,000 patients will die, ranking breast cancer second among cancer related deaths for women. When breast cancer cells express hormone receptors, including progesterone, estrogen, or Her2/neu receptors, there are several effective treatments targeting these receptors. Triple negative (TN) breast cancer, which comprises 15–20 % of breast cancer cells, lack these receptors and can aggressively invade and metastasize to distant organs. One current requirement for treating TN breast cancer is to develop therapeutic regimens that will maximize complete pathologic response rates to improve patient prognosis. Thus, the immediate requirements for treating TN breast cancer are to establish precise target therapies for patients by developing novel complexes that inhibit TN breast cancer invasion and metastasis in order to enhance patient response for improving patient outcomes.

There is substantial evidence demonstrating that metal-based reagents are promising candidates for cancer therapies. For example, complexes possessing platinum (Pt), such as cisplatin, carboplatin, and oxaliplatin, have been used to treat various cancer types such ovary, stomach, and colon [1, 2]. One of the mechanisms explaining how Pt complexes inhibit cancer cell growth is that they cause interstrand and intrastrand cross-linking of DNA, thereby inhibiting DNA repair or replication [3]. However, previous studies demonstrated that Pt complexes have severe side effects and generate resistant cancer cells, limiting the effectiveness of these complex in clinical practice [4]. Nevertheless, metal based reagents are promising anti-cancer drugs due to their ease of chemical modification and wide-spectrum of effectiveness against various origins of cancer.

Ru complexes are potent growth inhibitors for various cancer cells such as melanoma, ovarian, and breast [5–10]. Ru complexes have been proposed as an alternative to Pt complexes for development of novel anti-cancer drugs. Indeed, several Ru complexes are under phase I or II clinical trials [11–13]. Based on the structure-activity relationship studies, Ru complexes may function to inhibit tumor cells through mechanisms similar to that of cisplatin [14]. Some ruthenium (III) complexes (NAMI-A, KP1019 and KP1330) are in Phase II clinical trials [15]. In addition other organometallic ruthenium (II) arene complexes, RM175 and RAPTA complexes, have also shown promise [16, 17]. The nature of the ligands bound to the metal is important to the activity of the drug. In this study, we demonstrated that the Ru-arene complex [Ru(*η*^6^-*p*-cymene)(*o*-phenylendiamine)Cl]^+^ (*o*-PDA) is a potent anti-cancer reagent against breast cancer, osteosarcoma, lymphoma, and melanoma cells, while it did not affect cell growth of human epithelial cells, MCF10A. Using MDA-MB-231 cells, we demonstrated that *o*-PDA inhibited production of soluble growth factors such as VEGF-A, PDGF-AA, and GM-CSF at the transcriptional levels. Moreover, *o*-PDA and cyclophosphamide synergistically inhibited breast cancer cell growth. Thus, we provided information regarding the mechanisms of Ru-arene complex *o*-PDA to inhibit cancer cell growth, which supports synthesizing and testing additional Ru-arene complexes as novel anti-cancer agents.

## Methods

### Cell lines

Cell lines used in this study (MCF-10A, HCC38, SK-Br3, MCF-7, MDA-MB-231, HCC1806, Raji, Bowes, HT1080, and dermal fibroblasts) were purchased from ATCC. SUM149 cells were obtained from Dr. Soldano Ferrone (Massachusetts General Hospital, Boston, MA USA). MCF-10A cells were maintained in DMEM/F12 containing 5 % horse serum, 20 ng/ml EGF, 0.5 μg/ml hydrocortisone, 100 ng/ml cholera toxin, 10 μg/ml insulin, and 1 % penicillin/streptomycin. All other cell lines were cultured in RPMI640 containing 10 % FBS. Cells were cultured no more than 30 days after thaw in order to minimize phenotypic drift.

### Reagents

WST-1, doxorubicin, paclitaxel, and cyclophosphamide were purchased from CalBiochem (San Diego, CA, USA). Other reagents were purchased from Sigma-Aldrich (St. Louis, MO, USA) unless otherwise mentioned.

### Syntheses of Ru-complexes

The following reagents were obtained from Sigma-Aldrich: [(*p*-cymene)RuCl_2_]_2_ (*p*-cym), *o*-phenylenediamine (*o*-pda), and ammonium hexafluorophosphate (NH_4_PF_6_). Solvents were reagent grade. Benzene and diethyl ether were used as received. Methanol was distilled over iodine and magnesium.

#### [(*p*-cym)Ru(*o*-pda)Cl]PF_6_ (*o*-PDA) [6]

In a side-arm flask charged with nitrogen, [(η^6^-*p*-cym) RuCl_2_]_2_ (50.8 mg, 0.081 mmol) was suspended in 20 ml of freshly distilled methanol with stirring. After 5 min, *o*-pda (20.3 mg, 0.188 mmol) was added whereupon the deep orange solution immediately turned yellow. The solution was stirred at room temperature under nitrogen flow. After 30 min the solution was warmed in a water bath and the volume reduced to ½ under a steady flow of nitrogen; solid NH_4_PF_6_ (0.1205 g, 0.713 mmol) was added and the flask shaken to induce precipitation. The pale yellow precipitate was filtered and washed with diethyl ether and air-dried.

#### [(*p*-cym)Ru(*o*-bqdi)Cl]PF_6_ (*o*-BQDI) [6]

In a typical preparation [(η^6^-*p*-cym)RuCl_2_]_2_ (49.8 mg, 0.0813 mmol) and *o*-pda (21.0 mg, 0.184 mmol) were combined in 30 ml of freshly distilled methanol in a 125 ml Erlenmeyer flask and stirred in air at room temperature. The solution rapidly changed color from deep orange to dark purple. After 30 min, the volume was reduced to one-third by rotary evaporation and solid NH_4_PF_6_ (0.0608 g, 0.360 mmol) was added. Deep purple crystals formed after sitting in air overnight. The solid was recovered by vacuum filtration, rinsed with methanol and diethyl ether, and air-dried.

### Growth factor protein array study

MDA-MB-231 cells were seeded in 6-well plates in RPMI1640 containing 10 % FBS. Medium was replaced with serum-free RPMI1640 12 h before treatment with Ru complexes to minimize the residual effects on growth factor production by FBS-derived soluble factors. Cells were washed 4 times with serum-free RPMI1640 and incubated for 48 h with *o*-PDA and *o*-BQDI at a final concentration of 130 μM. Serum-free conditioned medium was collected and centrifuged for 3 min at 13.2 × 1000 rpm at room temperature to remove cell debris. The supernatant was immediately subjected to Human Growth Factor Antibody Array (Cat # AAH-GF-1, RayBiotech, Norcross, GA, USA) according to the manufacturer’s protocol. Briefly, antibody array was blocked for 30 min at room temperature with shaking and then incubated with 1 ml of conditioned medium at 4 °C overnight. The membranes were washed and incubated with biotinylated antibody cocktail at 4 °C overnight under constant shaking. The membranes were washed and the HRP-conjugated streptavidin was prepared and incubated with the membranes at 4 °C overnight under constant shaking. Finally, the membranes were washed and signals were detected using the detection reagents provided in the kit.

### Quantitative RT-PCR (qRT-PCR)

MDA-MB-231 cells were seeded in 10 mm tissue culture dishes. Cells were allowed to grow to ~80 % confluency before treatment with either *o*-PDA or *o*-BQDI at a final concentration of 130 μM. Cells were incubated at 37 °C under 5 % CO_2_ for 48 h. Cells were harvested from both medium and dishes by brief trypsinization. Total RNA was isolated by RNeasy Mini Kit protocol (QIAGEN, Redwood City, CA, USA). RNA was homogenized using QIAshredder (QIAGEN, Redwood City, CA, USA) and the first strand DNA was synthesized with High Capacity cDNA Reverse Transcription Kit according to manufacture protocol (Applied Biosystems, Foster City, CA, USA). PCR amplification was performed using TaqMan Universal PCR Master Mix (Applied Biosystems, Foster City, CA, USA). The TaqMan gene expression assays (Applied Biosystems, Foster City, CA, USA) used are as follows: PDGFA (Hs00964426_m1), GM-CSF (Hs00929873_m1), VEGF-A (Hs00900055_m1), and GAPDH (Hs02758991_g1) as reference gene. The reactions were first kept for 2 min at 50 °C followed by for 10 min at 95 °C. The cycling condition was 40 cycles of 15 s at 95 °C and 1 min at 60 °C. Quantification of gene expression was determined by Comparative C_t_ using ViiA 7 Real-Time PCR System (Applied Biosystems, Foster City, CA, USA).

### Statistical analysis

Two-tailed (paired) Student’s *t* test was used to calculate statistical significance between control and experimental groups. A *p* value of less than 0.001 is considered as significant difference between the groups.

### Growth assays

Cells were harvested with brief treatment with trypsin–EDTA and resuspended in RPMI-1640 containing 10 % FBS at a concentration of 1–2 × 10^5^ cells/ml. Cells (100 μl/well) were plated into 96-well plates and filled with an additional 100 μl/well of RPMI1640-10 % FBS. Cells were incubated at 37 °C under 5 % CO_2_ for 24 h, washed briefly, and incubated in the presence or absence of reagents at indicated concentrations for an additional 48 h. WST-1 (10 μl/well) was added to each well at the last 2 h of incubation and the absorbance at 450 nm was measured. As a control, the Ru complexes or anti-cancer reagents (i.e. puromycin) were incubated without cells for 48 h and incubated with WST-1 in order to compensate for the absorbance by these complexes. Cell growth was determined by measuring OD at 450 nm. Experiments were repeated at least three times with quadruplicate. Results were demonstrated as a mean % growth inhibition compared to control ± standard deviation (SD). EC50 was calculated according to the methods reported previously [18].

## Results

### Structural features of Ru-arene complexes used in this study

**Fig. 1:**
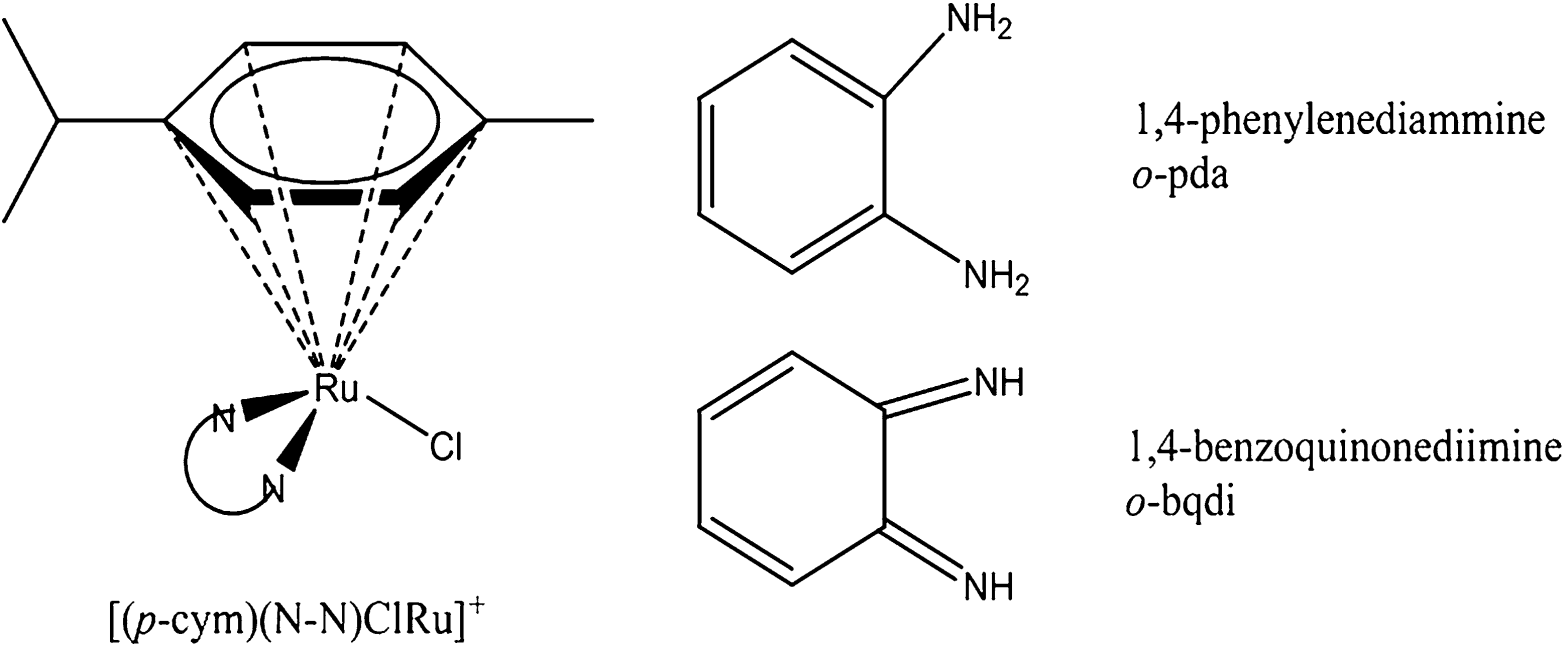
Structures of Ru-complexes used in this study. Structure of ruthenium complexes used in this study where N–N is either 1,2-phenylenediammine (*o*-pda) or 1,2-benzoquinonediimine (*o*-bqdi)

The complexes used in this study are shown in Fig. 1. They were prepared according to previously published procedures and characterized by UV–visible electronic absorption spectroscopy and ^1^H and ^13^C NMR. The spectral properties of the complexes agree with the values from the literature [6, 19]. The same starting materials were used to prepare both complexes. The *o*-PDA complex was prepared with freshly distilled methanol under nitrogen. When the procedure is carried out in air, ligand-based oxidation produces the diimine complex (*o*-BQDI). Chloride occupies the sixth binding site and the complexes are isolated as PF_6_^−^ salts. The complexes undergo hydrolysis in aqueous solution with water replacing the chloro ligand.

Ruthenium arene complexes of the general formula [Ru(η^6^-arene)(N–N)Cl]^+^, have three key structural features (Fig. 1): the arene ligand *para*-cymene (*p*-cym), a bidentate ligand with nitrogen as the donor atoms (N–N) and chloride as the other ligand. In this study, the N–N ligands are the aromatic diamine, *ortho*-phenylenediamine (*o*-PDA) or the oxidized form, *ortho*-benzoquinonediimine (*o*-BQDI). Binding studies of [(η^6^-arene)Ru(diamine)X]^+^ complexes show preferential binding between the metal and N7 of guanine bases on DNA [9]. The interaction is further stabilized through hydrogen bonding between the N–H of the amine and the C6O of the guanine [20, 21].

### Growth inhibition by Ru-arene complexes against various cancer cells

We first tested the growth inhibitory activity of the Ru-arene complexes for various cancer cells. Cells were incubated with various concentrations (0–260 μM) of *o*-PDA or *o*-BQDI for 48 h and then the growth was measured by colorimetric assays using WST-1. We tested human cells including breast cancer (HCC38, HCC1806, SKBr-3, MCF-7, and SUM149), B-cell lymphoma (Raji), osteosarcoma (HT1080), and melanoma (Bowes) (Additional file 1: Figure S1). Among the cell lines tested, MCF-7 (breast cancer, luminal A) and SK-Br-3 (breast cancer, Her2^+^) were most sensitive cells at EC_50_ of 50 μM of o-PDA (Table 1; Additional file 1: Figure S1). O-PDA inhibited growth of SUM149 (breast cancer, triple-negative), HT1080 (osteosarcoma), Raji (lymphoma), and Bowes (melanoma) at EC_50_ of 110 to 150 μM (Table 1; Additional file 1: Figure S1). Interestingly, growth of two breast cancer cell lines of triple-negative phenotype HCC38 and HCC1806 were not inhibited at the concentrations of o-PDA used in this study (Table 1; Additional file 1: Figure S1). On the other hand, o-BQDI did not show any growth inhibitory activity for cells except for SUM149 (Table 1; Additional file 1: Figure S1). Growth of SUM149 cells were inhibited at 40 % at the highest concentration (Additional file 1: Figure S1). The relatively higher EC_50_ obtained in this study compared other studies maybe due to the assay systems as direct counting cells and colorimetric assays using WST-1 [19]. Thus, these results suggest that o-PDA inhibited various cancer cell growth on a cell-type specific manner.

**Table 1:** Effects of RU-complexes on growth of various human cancer cells

Cell line^a^	EC50(µM)^b^	
	o-PDA	o-BQDI
HCC38 (breast ca. TN)	>260	>260
HCC1806 (breast ca. TN)	>260	>260
MCF-7 (breast ca. LA)	50	>260
SUM149 (breast ca. TN)	150	>260
HT1080 (osteosarcoma)	130	>260
Raji (lymphoma)	160	>260
Bowes (melanoma)	110	>260
SK-Br-3 (breast ca. Her2^+^)	50	>260

*TN* triple-negative, *LA* luminal A

^a^Cells (2 × 10^5^ cells/well) were treated serially diluted o-PDA or o-BQDI for 48 h. Cell growth was evaluated by colorimetric assays using WST-1 as an indicator. Experiments were repeated three times

^b^EC50 was calculated from three independent experiments. Standard error was less than 5 % of mean

In order to evaluate Ru-Arene complexes against metastatic breast cancer cells, we used MDA-MB-231 as a model system (Additional file 2: Figure S2). Cisplatin has been demonstrated as a potent anti-cancer agent against breast cancers [22]. *o*-PDA but not *o*-BQDI significantly inhibited growth of MDA-MB-231 cells was inhibited by o-PDA at EC_50_ = 83 μM (Table 2; Additional file 2: Figure S2). As previously reported [22], growth of MDA-MB-231 cells was inhibited in a concentration-dependent manner by cisplatin (IC_50_ = 53 μM) (Table 2; Additional file 2: Figure S2). MCF-10A cells are a spontaneously immortalized human epithelial cell line and show normal mammary epithelial cell morphology [23]. MCF-10A cells are used as a normal control in breast cancer studies [23]. When MCF-10A cells were incubated in the presence of Ru-Arene complexes, neither complex inhibited growth of cells at the concentrations tested (Table 2; Additional file 2: Figure S2), while growth was significantly inhibited even at 2.8 μM of cisplatin (Table 2; Additional file 1: Figure S1). These results suggest that *o*-PDA would be a potential therapeutic agent against metastatic breast cancer cells with minimal effect against breast epithelial cells.

**Table 2:** Effects of Ru-complexes on growth of MDA-MB-231 and MCF-10A cells

Cell line^a^	EC50(µM)^b^		
	o-PDA	o-BQDI	Cisplatin
MDA-MB-231	83	>260	53
MCF10A	>260	>260	2.8

^a^Cells (2 × 10 cells/well) were treated serially diluted o-PDA, o-BQDI or cisplatin for 48 h. Cell growth was evaluated by colorimetric assays using WST-1 as an indicator

^b^EC50 was calculated from three independent experiments. Standard error was less than 5 % of mean

### Inhibition of growth factor productions by *o*-PDA

**Fig. 2:**
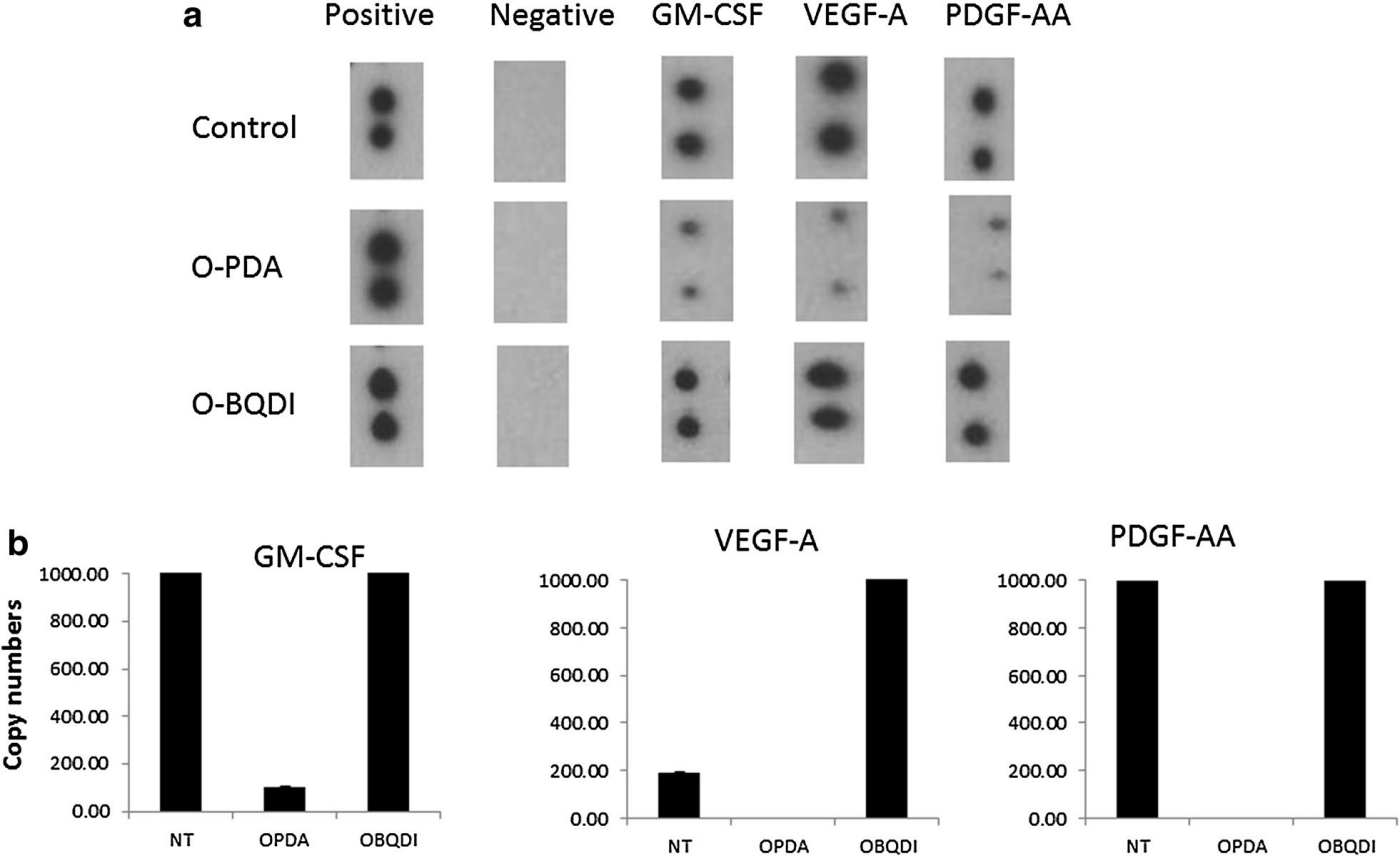
Inhibition of growth factor production from MDA_MB-231 cells treated with *o*-PDA. MDA-MB-231 cells were incubated in the presence of *o*-PDA or *o*-BQDI at a concentration of 130 μM for 48 h in serum-free RPMI1640. **a** Conditioned medium was harvested, centrifuged, and subjected to Human Growth Factor Array. **b** Cells were harvested and total RNA was purified, transcribed, and then subjected to RT-PCR reactions using specific primer sets for GM-CSF, VEGF-A, and PDGF-AA

In order to ask whether *o*-PDA could have potential effects on breast cancer cells which could confer progression and metastasis, we analyzed the production of soluble factors from MDA-MB-231 cells, Cells were incubated with 130 μM of *o*-PDA or *o*-BQDI for 48 h and the conditioned medium was subjected to growth factor protein array. The production of PDGF-AA, VEGF-A, and GM-CSF was markedly reduced when cells were incubated in the presence of *o*-PDA but not with *o*-BQDI (Fig. 2a). In order to test whether the inhibition of the proteins of growth factors would be the result of transcriptional regulation, we performed quantitative RT-PCR (qRT-PCR) analyses to estimate copy numbers of these growth factors from MDA-MB-231 cells treated with *o*-PDA, and *o*-BQDI. These results demonstrated that treatment with *o*-PDA markedly reduced copy numbers of transcripts of PDGF-AA, VEGF-A, and GM-CSF compared to the control and *o*-BQDI-treated cells (Fig. 2b), suggesting that *o*-PDA reduces the production of specific growth factors in MDA-MB-231 cells at the transcriptional levels.

### Growth inhibition of breast cancer cells by combination of *o*-PDA and chemotherapeutic agents

**Fig. 3:**
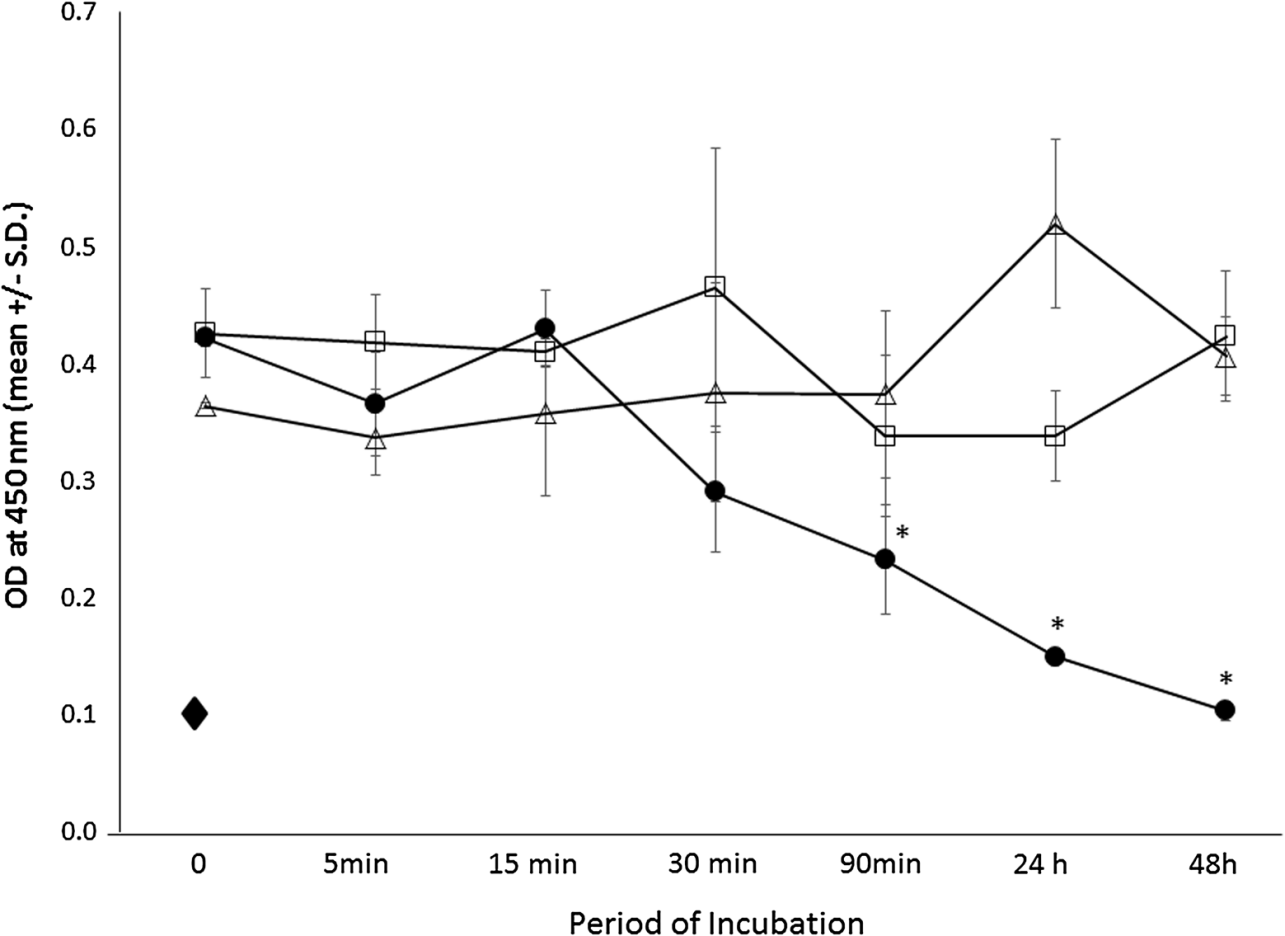
Inhibition of cell growth by *o*-PDA and cyclophosphamide. MDA-MB-231 cells (1.25 × 10^4^ cells/well) were incubated over various time periods in the presence of cyclophosphamide alone (10 mM) (), *o*-PDA alone (32 μΜ) (), or the combination of the two reagents () for up to 48 h. Cells were incubated with Puromycin (25 mM) for 48 h (). Cell growth was evaluated by colorimetric assays using WST- as an indicator. Experiments were repeated three times. Results were demonstrated as a mean ± SD of OD_450_. *p < 0.001 (calculated by Student’s two-tailed *t* test)

In order to test whether *o*-PDA would enhance the cytotoxic activity of chemotherapeutic agents, MDA-MB-231 cells were incubated in the presence of suboptimal concentrations of *o*-PDA (32 μM) and cyclophosphamide (10 mM) for various time intervals from 5 min to 48 h (Fig. 3). Cyclophosphamide inhibited MDA-MB-231 cell growth at the same concentration range reported previously [24]. When cells were incubated up to 48 h in the presence of both reagents, a significant growth inhibition was observed in a time-dependent manner compared to single treatments. This synergistic growth inhibition was observed when cells were incubated for as little as 90 min (Fig. 3). These results suggest that *o*-PDA and cyclophosphamide synergistically inhibited breast cancer cell growth in a time-dependent manner.

**Fig. 4:**
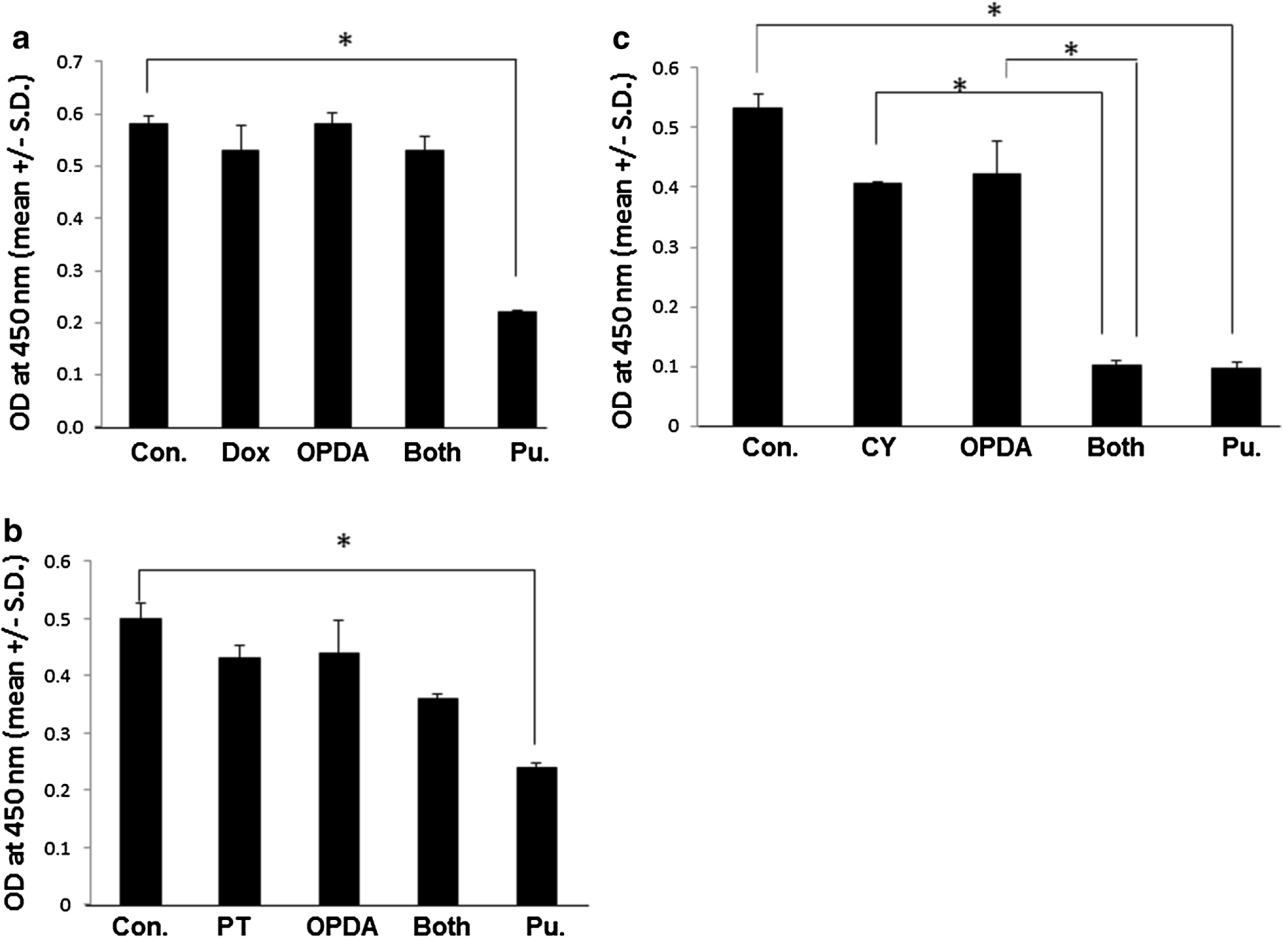
Inhibition of cell growth by the combination of *o*-PDA with other cytotoxic agents. MDA-MB-231 cells (1.25 × 10^4^ cells/well) were incubated for 2 days in the presence of Doxorubicin (DOX, 5 μM) (**a**), Paclitaxel (PT 5 μM) (**b**) or cyclophosphamide (CY 10 mM) (**c**) with or without *o*-PDA (32 μΜ). Puromycin (25 mM) was used as a positive control. Cell growth was evaluated by colorimetric assays using WST-1 as an indicator. Experiments were repeated three times. Results were demonstrated as a mean ± SD of OD_450_. *p < 0.001 (calculated by Student’s two-tailed *t* test)

On the other hand, when cells were incubated in the presence of *o*-PDA and doxorubicin or paclitaxel, no significant synergistic growth inhibition was observed (Fig. 4). These results suggest specific mechanisms of growth inhibition of *o*-PDA and cyclophosphamide.

## Discussion

It has been suggested that several unique features of ruthenium (Ru)-arene complexes would be beneficial for developing anti-cancer drugs. One is the ease of chemical structure modification by substituting different arene ligands and the bidentate O- and N- donor ligands. Another is the design complexes that will bind to cell surface receptors such as transferrin receptor (CD71) or integrins [25, 26]. In this study, we demonstrated that *o*-PDA significantly inhibited not only breast cancer cells but also other cancer cell types including osteosarcoma, melanoma, and lymphoma. Additionally, it did not affect normal epithelial (MCF-10A) cell growth. Using MDA-MB-231 cells as a model system, we demonstrated that *o*-PDA inhibited the production of critical growth factors such as PDGF-AA, GM-CSF, and VEGF-A at the transcriptional levels. Importantly, combining suboptimal concentrations of *o*-PDA and cyclophosphamide enhanced cytocidal activity in MDA-MB-231 cells. These results suggest that Ru-Arene complexes are potential anti-cancer reagents *per se* in monotherapy as well as in combination with neoadjuvants such as cyclophosphamide.

Sadler and co-workers observed cell-type specific growth inhibition by *o*-PDA [8, 27]. In this study, we explored various cell lines for their sensitivities against this complex. Growth of melanoma, lymphoma, and osteosarcoma was significantly inhibited by *o*-PDA. Among breast cancer cells, growth of Her2^+^ (SK-Br-3), luminal A (MCF-7), and triple-negative (MDA-MB-231) was inhibited in the presence of *o*-PDA in a concentration-dependent manner. However, other triple-negative breast cancer cells, HCC38 and HCC1806, were resistant to this complex. There is insufficient information to understand the cell type-specific growth inhibition by *o*-PDA at present. Extensive structure-activity studies have shown that all three components (arene ligand, N–N donor ligand and chloride) are important to cytotoxicity of Ru complexes [8, 9, 27–29]. More specifically, cytotoxic behavior is not observed (high IC_50_) in [(η^6^-arene)Ru(N–N)Cl]^+^ complexes which cannot form NH-C6O hydrogen bonds [8]. Computational studies of the 9-ethylguanine adduct of *o*-PDA shows Ru binding to N7 with hydrogen bonding between C6O of the guanine and the coordinated *o*-PDA. The planar structure of the oxidized *o*-bqdi ligand imparts rigidity resulting in a greater distance between the NH protons and a much weaker hydrogen bond to C6O [27]. Adhireksan et al. [30] performed a very detailed structure-activity relationship study of two Ru-arene complexes on cell growth inhibition and demonstrated that a cytotoxic Ru-arene complex targets the DNA of chromatin, while a non-cytotoxic complex forms adducts within the histone proteins. This is an attractive hypothesis which may explain the cell-type specific growth inhibition by Ru-arene complexes. While cisplatin significantly inhibited normal human epithelial cells, MCF-10A, this cell line was resistant against the treatment with *o*-PDA. These results suggest that Ru-Arene complexes such as *o*-PDA would be attractive anti-cancer reagents with minimal growth inhibitory activity against breast epithelial cells.

Previous studies demonstrated that soluble factors produced from malignant tumor cells would alter tumor/tissue microenvironments favoring tumor growth and invasion into surrounding tissues. For example, the production of PDGF-A is significantly associated with lymph node metastasis of breast cancer cells [31]. Furthermore, PDGF-A and its receptor PDGF-α expressions on the same breast cancer cells suggest that PDGF-A/PDGF-α loop would function as an autocrine growth mechanism [32]. Importantly, previous studies demonstrated that neovascularization surrounding tumor mass is a critical process for facilitating progression and metastasis. Indeed, it was reported that the expression of VEGF-A is associated with shorter survival times with triple negative breast cancer patients [33]. These results suggest that targeting VEGF-A may be an alternative way to improve outcomes in patients who are diagnosed with triple-negative phenotype. Breast cancer cells tend to metastasize to bone and modulate the biological functions of bone cells. Utilizing MDA-MB-231 cells, Mendoza-Villanueva et al. [34] reported that GM-CSF and IL-11 play a key role in inducing differentiation of osteoblasts. Thus, it is anticipated that inhibition of GM-CSF from breast cancer cells may have an impact on the bone cell functions such as osteoblasts at the metastasized lesion. In this study, we demonstrated that inhibiting the production of VEGF-A, PDGF-AA, and GM-CSF by treatment with *o*-PDA would lead to an efficient blockade of tumor growth and osteoblasts functions at bone.

The regulation of VEGF-A protein production is mediated by multiple pathways. For example, it is reported that endoplasmic reticulum ER-associated degradation pathways are key processes for degrading unassembled subunits of multimeric proteins [35]. Vesicles containing VEGF-A molecules are transported through the ER-Golgi apparatus, in which they become encapsulated in vesicles. These vesicles may be subjected to degradation through ubiquitination followed by degradation of proteasomes, thereby degrading VEGF-A in the cytoplasm prior to exocytosis [36]. Thus, one explanation for the discrepancy of the production of VEGF-A protein and mRNA in MDA-MB-231 cells treated with or without o-BQDI is the possibility that the production of VEGF-A protein could be regulated on the post-translational level including ubiquitination-proteasome systems for producing appropriate amounts of VEGF-A, which is an analogy for regulation of transcription factors [37]. As an alternative explanation, it might be possible that o-BQDI would prevent production of VEGF-A from cells by attenuating cytoplasmic translocation and/or bursting of VEGF-A-containing vesicles by altering intracellular pH [38]. Indeed, previous studies demonstrated that a ruthenium compound changed intracellular pH in neuron cells [39]. Therefore, it would be important to characterize mechanisms of regulation of intracellular environment by anti-cancer drugs (i.e. Pt and Ru compounds) for developing therapeutic strategies for cancer patients.

Recent studies suggest that multiple modulating strategies in combination with chemotherapeutic reagents would be promising approaches for improved treatment of breast cancer [40]. In order to maximize therapeutic efficacy while decreasing the side effects of the reagents, there is a need to develop molecules that synergistically inhibit cancer cell growth with chemotherapeutic reagents. We demonstrated that *o*-PDA synergistically inhibited MDA-MB-231 cells with cyclophosphamide. Clinically, cyclophosphamide has been used as an effective chemotherapeutic agent for breast cancer patients [41]. Cyclophosphamide is a alkylating reagent that attaches to the N7 of guanine and forms interstrand and intrastrand DNA crosslinks [42]. As discussed above, *o*-PDA will bind to N7 of guanine through Ru and the NH of the *o*-PDA ligand forms hydrogen bonds with the carbonyl oxygen of carbon 6, thereby inducing premature termination of RNA synthesis [20, 21, 28, 43]. Thus, it is postulated that targeting N7 of guanine would be one of the mechanisms of the observed synergistic effect by *o*-PDA and cyclophosphamide to inhibit MDA-MB-231 cell growth. Treatment of mice bearing melanoma with cyclophosphamide induces immunosuppression in an inflammation-dependent manner and impaired anti-tumor effect in vivo [44]. Thus, it is expected that decreasing the treatment dose of cyclophosphamide with co-administration of *o*-PDA would be beneficial toward increasing direct cytocidal activity to cancer cells while decreasing its immunosuppressive effect. Recent studies demonstrated that treatment of cancer cells such as colon with cyclophosphamide increases the number of colon cancer stem cells [45].

In summary, we demonstrated the efficacy of *o*-PDA as a potent growth inhibitor for tumor cells but not normal epithelial cells. There are several issues that remain to be addressed regarding the mechanisms of cell growth inhibition by *o*-PDA such as the presence of cell surface receptors for *o*-PDA and the mechanisms of cell growth inhibition. Further systematic and extensive structure-activity relationship studies are imperative for developing new Ru-arene complexes that act as effective chemotherapeutic reagents, which may prevent invasion and metastasis with inhibition of multiple processes of tumor growth.

## Conclusions

We demonstrated that treatment of MDA-MB-231 cells with *o*-PDA inhibited growth factor productions such as VEGF-A, PDGF-AA, and GM-CSF. *o*-PDA and cyclophosphamide synergistically inhibited growth of MDA-MB-231 cells. These results suggest that Ru-arene complexes are promising anti-cancer reagents by inhibiting multiple processes of tumor cell growth.

## Additional files

10.1186/s12967-016-0797-9 Inhibition of cell growth by Ru-arene complexes. Various cells including HCC38 (breast cancer), HCC1806 (breast cancer), SK-Br3 (breast cancer), MCF-7 (breast cancer), SUM149 (breast cancer), Raji (B-cell lymphoma), HT1080 (osteosarcoma), and Bowes (melanoma) cells (2 × 10^5^ cells/well) were incubated in the presence of *o*-PDA () or *o*-BQDI (), at various concentrations for 2 days. Dashed line represents the growth inhibition by incubating with Puromycin (25 mM). Cell growth was evaluated by colorimetric assays using WST-1 as an indicator. Experiments were repeated three times. Results were demonstrated as a mean % of inhibition compared to control mean ± standard deviation (SD). OD_450_ of control cells was 0.4 to 0.5 in each cell line. **p* < 0.001 (calculated by Student’s two-tailed *t*-test).
10.1186/s12967-016-0797-9 Growth inhibition of MDA-MB-231 and MCF-10A cells by Ru-arene complexes. (A) MDA-MB-231 cells (2 X 10^5^ cells/well) were incubated in the presence of *o*-PDA or *o*-BQDI (A) or cisplatin (B) at various concentrations for 2 days. MCF-10A cells (2 X 10^5^ cells/well) were incubated in the presence of *o*-PDA, or *o*-BQDI at various concentrations (C) or cisplatin (D) for 2 days. Cell growth was evaluated by colorimetric assays using WST-1 as an indicator. Symbols: *o*-PDA (), *o*-BQDI (). Dashed line represents the growth inhibition by incubating with Puromycin (25 mM). Experiments were repeated three times. Results were demonstrated as a mean  % of inhibition compared to control mean ± standard deviation (SD). OD_450_ of control cells was 0.4 to 0.5 in each cell line. **p* < 0.001 (calculated by Student’s two-tailed *t*-test).
