# Supplementary figures and images for: Inhibition of cancer cell growth by ruthenium complexes
Source: J Transl Med. 2016 Feb 12;14:48. doi: 10.1186/s12967-016-0797-9 (PMC4751662; doi:10.1186/s12967-016-0797-9)

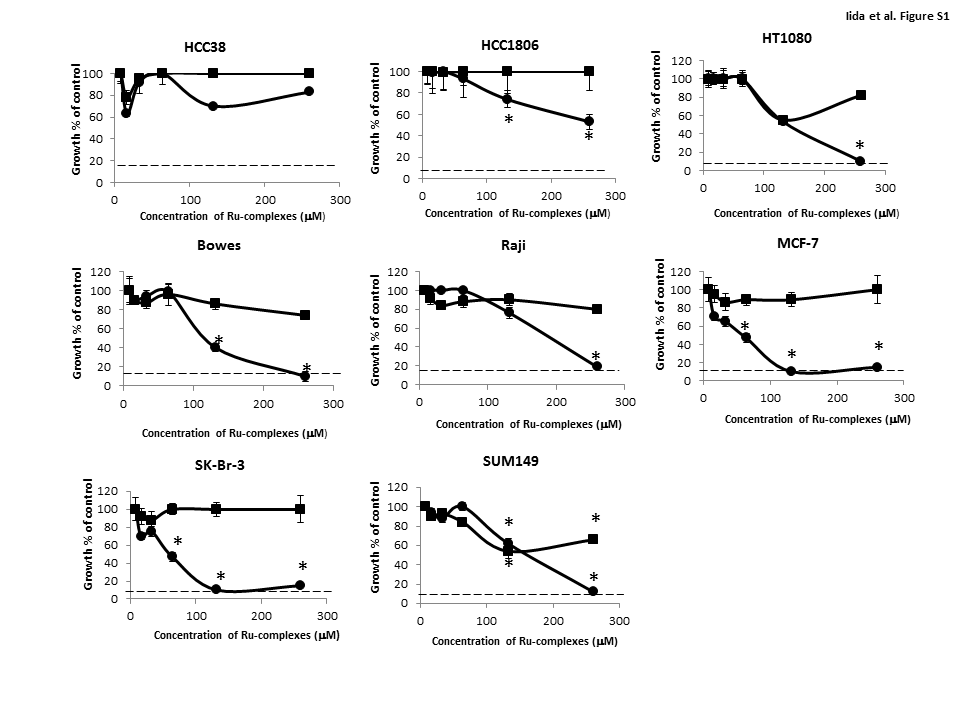

Supplement: Supplementary file 1 — 10.1186/s12967-016-0797-9 Inhibition of cell growth by Ru-arene complexes. Various cells including HCC38 (breast cancer), HCC1806 (breast cancer), SK-Br3 (breast cancer), MCF-7 (breast cancer), SUM149 (breast cancer), Raji (B-cell lymphoma), HT1080 (osteosarcoma), and Bowes (melanoma) cells (2 × 10^5^ cells/well) were incubated in the presence of o-PDA () or o-BQDI (), at various concentrations for 2 days. Dashed line represents the growth inhibition by incubating with Puromycin (25 mM). Cell growth was evaluated by colorimetric assays using WST-1 as an indicator. Experiments were repeated three times. Results were demonstrated as a mean % of inhibition compared to control mean ± standard deviation (SD). OD450 of control cells was 0.4 to 0.5 in each cell line. *p < 0.001 (calculated by Student’s two-tailed t-test). [file 12967_2016_797_MOESM1_ESM.tif]

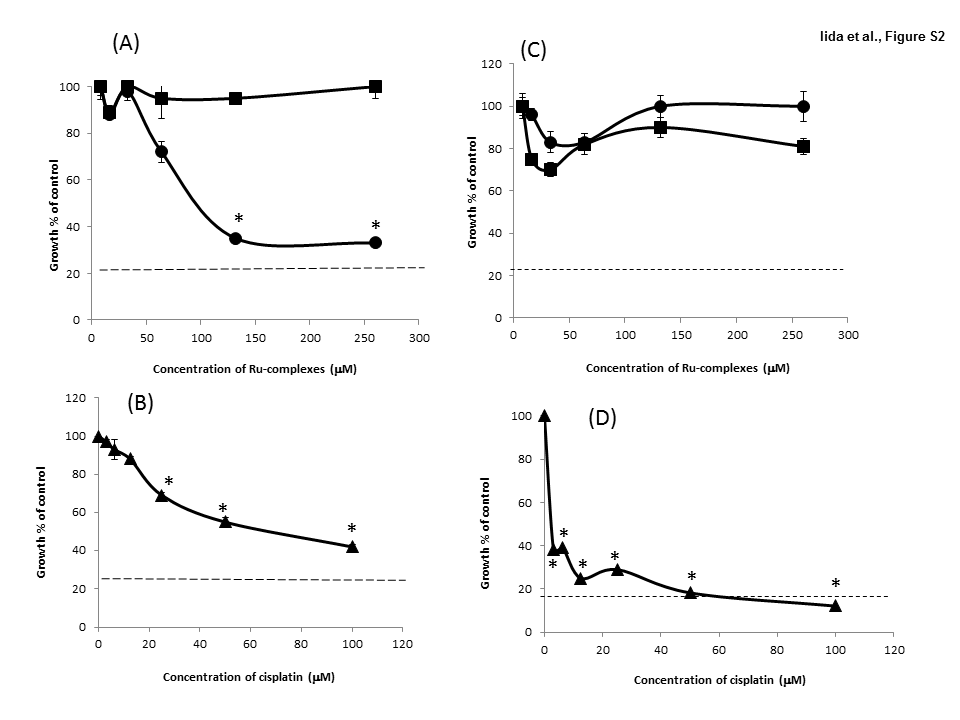

Supplement: Supplementary file 2 — 10.1186/s12967-016-0797-9 Growth inhibition of MDA-MB-231 and MCF-10A cells by Ru-arene complexes. (A) MDA-MB-231 cells (2 X 105 cells/well) were incubated in the presence of o-PDA or o-BQDI (A) or cisplatin (B) at various concentrations for 2 days. MCF-10A cells (2 X 105 cells/well) were incubated in the presence of o-PDA, or o-BQDI at various concentrations (C) or cisplatin (D) for 2 days. Cell growth was evaluated by colorimetric assays using WST-1 as an indicator. Symbols: o-PDA (), o-BQDI (). Dashed line represents the growth inhibition by incubating with Puromycin (25 mM). Experiments were repeated three times. Results were demonstrated as a mean % of inhibition compared to control mean ± standard deviation (SD). OD450 of control cells was 0.4 to 0.5 in each cell line. *p < 0.001 (calculated by Student’s two-tailed t-test). [file 12967_2016_797_MOESM2_ESM.tif]
